# Beta band oscillations in the motor thalamus are modulated by visuomotor coordination in essential tremor patients

**DOI:** 10.3389/fnhum.2023.1082196

**Published:** 2023-04-26

**Authors:** Diellor Basha, Suneil K. Kalia, Mojgan Hodaie, Adriana L. Lopez Rios, Andres M. Lozano, William D. Hutchison

**Affiliations:** ^1^Department of Physiology, University of Toronto, Toronto, ON, Canada; ^2^Division of Clinical and Computational Neuroscience, Krembil Research Institute, Toronto, ON, Canada; ^3^Division of Neurosurgery, Toronto Western Hospital, Toronto, ON, Canada; ^4^Department of Surgery, University of Toronto, Toronto, ON, Canada; ^5^Hospital Universitario de San Vicente Fundación, Medellín, Colombia; ^6^Hospital de San Vicente Fundación, Rionegro, Colombia

**Keywords:** intraoperative microrecording, basal ganglia, motor thalamus, beta oscillation, essential tremor

## Abstract

**Introduction:**

Beta oscillations in sensorimotor structures contribute to the planning, sequencing, and stopping of movements, functions that are typically associated with the role of the basal ganglia. The presence of beta oscillations (13–30 Hz) in the cerebellar zone of the thalamus (the ventral intermediate nucleus – Vim) indicates that this rhythm may also be involved in cerebellar functions such as motor learning and visuomotor adaptation.

**Methods:**

To investigate the possible role of Vim beta oscillations in visuomotor coordination, we recorded local field potential (LFP) and multiunit activity from the Vim of essential tremor (ET) patients during neurosurgery for the implantation of deep brain stimulation (DBS) electrodes. Using a computer, patients performed a visuomotor adaptation task that required coordinating center-out movements with incongruent visual feedback imposed by inversion of the computer display.

**Results:**

The results show that, in ET, Vim beta oscillations of the LFP were lower during the incongruent center-out task than during the congruent orientation. Vim firing rates increased significantly during periods of low beta power, particularly on approach to the peripheral target. In contrast, beta power in the subthalamic nucleus of Parkinson’s disease (PD) patients did not differ significantly between the incongruent and the congruent orientation of the center-out task.

**Discussion:**

The findings support the hypothesis that beta oscillations of the Vim are modulated by novel visuomotor tasks. The inverse relationship between the power of Vim-LFP beta oscillations and Vim firing rates suggest that the suppression of beta oscillations may facilitate information throughput to the thalamocortical circuit by modulation of Vim firing rates.

## 1. Introduction

Essential tremor (ET) is the most common movement disorder, characterized by tremor symptoms that arise during kinetic, postural or isometric motor activity (Bain et al., 2000). While the histopathology of ET is still debated, it is evident that some form of cerebellar dysfunction is involved in the manifestation of tremor (Louis et al., 2007; Deuschl and Elble, 2009). A proportion of ET patients exhibit deficits in tandem gait (Stolze et al., 2001; Fasano et al., 2010) and show marked impairment in cerebellar functions such as hand-eye coordination and visuomotor adaptation (Schwartz et al., 1999; Trillenberg et al., 2006). Deep brain stimulation (DBS) or ablative surgical intervention in the cerebellar zone of the thalamus is consistently effective against ET (Flora et al., 2010) although some adverse effects of DBS include dyspraxia in 0.47% of cases and incoordination in 1.4% of cases, suggesting that the integrity of the cerebellar thalamus is required for effective visuomotor coordination. Consistent with this idea, Chen et al. (2006) found that DBS stimulation of the Vim affects motor adaptation in a voltage-dependent manner and that lesioning the Vim impairs motor adaptation in the contralateral arm.

The acquisition of complex motor skills occurs through motor adaptation and motor sequence learning (Martinu and Monchi, 2013). It is commonly thought that motor adaptation and motor sequences are mediated through anatomically and functionally separate mechanisms. Motor adaptation, the ability to compensate for changing environments, is thought to occur largely through error-based learning mediated by the cerebellar system (Doyon et al., 2003). Consistent with this idea, studies in human subjects and in animal models show that the activity of the cerebellar cortex and the dentate nucleus correlates with corrective movements following perturbation in sensory feedback (Tseng et al., 2007; Koziol et al., 2014). Patients with hereditary or acquired cerebellar damage have impairment in visuomotor adaptation tasks, such as when visual misalignment is imposed by prism goggles (Weiner et al., 1983). Motor sequences, such as the playing of sequential notes in a musical phrase, are mediated by the basal ganglia and depend on reinforcement-learning and dopaminergic mechanisms. In the STN (Herrojo Ruiz et al., 2014) and the globus pallidus (Herrojo Ruiz et al., 2014), motor sequence learning is coded by transient, event-related desynchronizations (ERDs) of beta oscillations occurring around a sequence of movements rather its constituent elements. Excessive beta power and impaired ERD in the basal ganglia are linked to anti-kinetic PD symptoms and are dependent on dopaminergic medication (Levy et al., 2002). Contrary to the traditional view of the cerebellum and basal ganglia as parallel processing channels, the discovery of bidirectional connections between the two systems (Hoshi et al., 2005; Bostan et al., 2010) suggests motor adaptation and sequencing may be more closely integrated than previously thought. Some studies have linked PD pathology to visuomotor adaptation (Mongeon et al., 2013; Singh et al., 2019), reporting that unmedicated PD is associated with visuomotor deficits. Imaging studies have also implicated the basal ganglia in visuomotor adaptation (Seidler et al., 2006; Tan et al., 2014). Considering the link between PD pathology, OFF-medication states and beta oscillations in the subthalamic nucleus (Levy et al., 2002), we considered the possibility that STN beta oscillations contribute to visuomotor coordination and are modulated by visuomotor tasks.

The magnitude of beta ERD depends on the subject’s familiarity with the motor task being performed suggesting that beta ERD may reflect the level of expertise in learned movements (Kilavik et al., 2013). In the early stages of learning, when a subject is unfamiliar with the sensorimotor dynamics of the task, the large suppression of beta is correlated with superior performance (Pollok et al., 2014). Fast cortical rhythms such as beta are thought to facilitate motor learning by providing a temporal link between sensory and motor centers (Bibbig et al., 2002). This allows the brain to register the sensory consequences of a movement in real time and evaluate sensory results against the motor command. Unexpected sensory stimuli produce a large suppression of beta power whereas expected sensory stimuli produce small beta suppression (Pavlidou et al., 2014). The post-movement beta rebound has also been suggested to reinforce existing motor states and steady motor output (Engel and Fries, 2010) and appears to be involved in the processing of movement-related sensory afference (Alegre et al., 2008) and in errors related to the completed movement (Tan et al., 2014). Thus, a familiar task such as the manipulation of a computer mouse may produce low beta suppression considering that the mouse cursor (visual feedback) responds predictably to the learned movement of the hand (motor command). However, if the cursor moves in the opposite direction to the hand movement, some form of adaptation is required to learn the novel visuomotor relationship.

In a previous electrophysiological study of the Vim (Basha et al., 2014), we found that a prominent beta oscillations were localized to the Vim and were desynchronized by movement, indicating its involvement in motor control. However, whether this Vim beta rhythm relates to cerebellar function and whether it is involved in motor adaptation is yet unknown. Based on previous findings linking cortical beta ERD with visuomotor adaptation, we hypothesized that the suppression of Vim beta power is modulated by novel visuomotor tasks. Considering the possible involvement of the basal ganglia in visuomotor coordination, we hypothesized that subthalamic beta oscillations are modulated by visuomotor tasks.

## 2. Methods

### 2.1. Patients

A total of 19 patients participated in the experiments. The ET groups consisted of 10 ET patients (mean age 58.5 ± SD 11, 4 female) and 9 PD patients (mean age 59.7 ± SD 5.0, 4 female, Table 1). Pre-operative clinical assessment of all patients was performed by a neurologist at Toronto Western Hospital using the Unified Parkinson’s Disease Rating Scale (UPDRS) for PD patients and the Fahn-Tolosa-Marin tremor scale (Fahn, 1987) ET patients. Recordings were obtained from awake patients under local anesthesia who were withdrawn from all medications 12 h prior to surgery. During recording, all ET patients presented with action tremor of varying intensity, contralateral to the side of recordings. All PD patients presented with resting tremor during the surgery. Patients with exaggerated tremor that were unable to complete more than five trials of the hand-eye coordination experiments were excluded from the study. See Table 1 for clinical and demographic details of the patients. The protocol used in these studies was reviewed and approved by the University Health Network Ethical Review Board (REB07-5006.01). All patients gave free and informed consent to participate in the study.

**Table 1:** Demographic and clinical characteristics of patients.

Patient	Age/Sex	DBS target	Distance from target (mm)	Medications
ET 1	55 M	Left Vim	2.3	Lorazepam 1 mg
ET 2	76 M	Right Vim	4	Coversyl 8 mg QAM
				Divaloproex-250,750 mg QAM
				Clonazepam 0.25 mg PRN
ET 3	41 F	Left Vim	N/A	N/A
ET 4	65 M	Left Vim	6.8	Lorazepam 1 mg
ET 5	58 F	Left Vim	5.8	Primidone 125 mg
ET 6	55 F	Left Vim	5.8	Primidone 125 mg
ET 7	47 F	Right Vim	5	Lorazepam 1 mg
ET 8	63 M	Left Vim	7.2	Lorazepam 1 mg
ET 9	75 M	Left Vim	N/A	N/A
ET 10	50 M	Left Vim	8	Lorazepam 1 mg
PD 1	51 M	Bilateral STN	1.9	Levodopa/carbidopa 100/25 2 tabs/day
				Levodopa/carbidopa/entacapone as directed
				Quietapine 75 mg QHS
				Paroxetine 30 mg QHS
PD 2	59 M	Bilateral STN	1.5	Levodopa/carbidopa 100/25 2 tabs as directed
				Entacapone 200 mg as directed
				Aggrenox 1 ttab BID
				Florinef 0.2 mg QAM
PD 3	62 F	Bilateral STN	0.8	Levodopa/carbidopa 100/25 1 tab/day
				Ropinirol 2 mg TID
				Trazodone 50 mg QHS
				Remeron 15 mg QHS
				Ativan 0.5 mg QHS
PD 4	68 F	Bilateral STN	3.2	Levodopa/carbidopa 100/25 2 tabs/day
				Entacapone 200 mg as directed
PD 5	58 M	Bilateral STN	1	Levodopa/carbidopa 100/25 as directed
				Domperidone 60 mg/day
PD 6	64 M	Bilateral STN	0.8	Levodopa/carbidopa 100/25 1 tab/day
				Amantadine 100 mg 1/day
				Citalopram 10 mg qPM
PD 7	55 F	Bilateral STN	1.5	Levodopa/carbidopa 100/25 1 tab/day
				Prolopa CAP 200–50 1 tab as directed
				Entacapone 200 mg as directed
				Clonazepam 0.5 mg as directed
PD 8	63 M	Bilateral STN	2.2	Levodopa/carbidopa 100/25 BID
				Pregabalin 75 mg BID
				Clonazepam 0.5 mg QHS
PD 9	58 F	Bilateral STN	4	Levodopa/carbidopa 100/25 2 tabs/day
				Clonazepam 1 mg

### 2.2. Recordings

**Figure 1:**
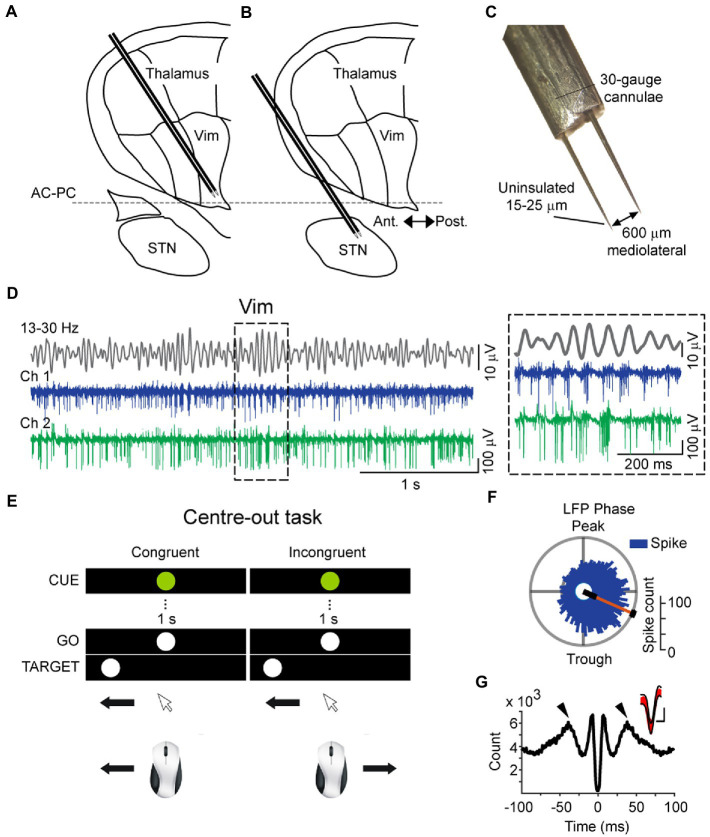
Schematic of intraoperative microelectrode recordings of the thalamus **(A)** and subthalamic nucleus **(B)**, superimposed on a sagittal Schaltenbrand and Wahren map, 14.5 mm from the midline. **(C)** Dimensions of dual microelectrodes used for intraoperative recordings, visualized in 10× magnification. **(D)** Recording traces of the cerebellar zone of the thalamus (ventral intermediate nucleus – Vim) obtained simultaneously from two microelectrodes (Ch1 and Ch 2) and the bandpassed signal from Ch1 (top, 13–30 Hz), showing the modulation of beta amplitude and corresponding spiking activity. Inset: example recordings showing the coupling of Vim multi-unit activity to the phase of the field beta oscillation. **(E)** Schematic of the center-out task conducted by patients during intraoperative microelectrode recordings. Patients were instructed to aim toward the peripheral target after a brief visual fixation in two conditions: “normal” orientation with congruent hand-eye orientation and incongruent (“inverted”) orientation. **(F)** Vim single-units were phase-locked to the rising phase of the LFP beta oscillation (Rayleigh Uniformity test, pRayleigh < 0.001). **(G)** Spike-triggered average of the Vim field potential, showing rhythmic peaks at the beta cycle (50 ms) and the rising phase of the field around the time of the spike. The large negative deflection at 0 ms is due to the averaged negative polarity of multi-unit activity. Inset: overlaid spike waveforms (red) and the confidence boundaries used during template matching in spike detection.

Spike and local field potential (LFP) recordings of the Vim (ET patients) or STN (PD patients) nuclei were obtained during electrophysiological mapping procedures in DBS surgery. Two microelectrodes (Figures 1A–D) about 25 μm tip length, axes 600 μm apart, about 0.2-MΩ impedance at 1,000 Hz) were inserted into the thalamus or STN of the awake patient to physiologically localize the surgical target in conjunction with imaging procedures. The localization procedure for the Vim using microelectrodes has been described elsewhere in detail (Tasker et al., 1997). Briefly, the stereotactic coordinates of the anterior commissure (AC) and the posterior commissure (PC) were first determined by 1.5 T or 3 T magnetic resonance imaging and were used as landmarks for the estimated localization the ventral thalamic nuclear group according the 14.5 mm sagittal section of the stereotactic atlas of Schaltenbrand and Wahren (1977). For the electrophysiological identification of the Vim, microstimulation (100 μA, 200 Hz, 1 s, pulse width 0.3 s) was conducted every 1 millimeter along the trajectory of the microelectrode and stimulation-induced tremor reduction or arrest were observed and semi-quantitatively assessed by visual inspection and also by the reduction in amplitude of the accelerometer tracing obtain from the wrist. Because the Vim is bordered in its posterior extent by the somatosensory thalamus (ventral caudal- Vc), the first site in the trajectory where somatosensory effects were evoked by microstimulation were taken to indicate the posterior border of Vim. In accordance with the Schaltenbrand and Wahren atlas (1977), the area within 3 mm anterior to this border was defined as the Vim nucleus. Second, single- or multi-unit activity that was modulated by passive movements of the contralateral limb was noted as an electrophysiological signature of Vim motor thalamic neurons (Molnar et al., 2005). Third, the most efficacious tremor-reduction or arrest in response to microstimulation (as noted above) along the trajectory was taken to indicate a Vim site. Fourth, an increase in beta oscillatory activity in the single-unit and LFP signals detected online by active fast Fourier transform of the signal (Spike 2, Cambridge Electronic Design, United Kingdom) was used to identify Vim activity (Basha et al., 2014). In the PD group of patients, recordings were collected from the dorsal sensorimotor partition of the STN nucleus which was identified according to microelectrode-guided targeting procedures described previously by Hutchison et al. (1998) (Figure 1B). Briefly, the dorsolateral STN was identified by an increase in background activity and high-frequency neuronal discharge in the beta frequency range. A decrease in background activity relative to the dorsal STN indicated entry into the ventral portion in the STN. In the ventroposterior progression of the electrode, the substantia nigra par reticulata was identified after the STN by higher-frequency (90 Hz), regular and low-amplitude discharges. Recordings were fine-tuned by moving the microelectrodes in order to obtain stable single-unit recordings in at least one of the two microelectrodes. All recordings were amplified 5,000–10,000 times, filtered at 10–5,000 Hz (analog Butterworth filters: high pass, 1 pole; low pas, 2 poles) using two Guideline System Neuroamp- 1A amplifiers (Axon Instruments, Union City, CA).

### 2.3. The center-out task

**Figure 2:**
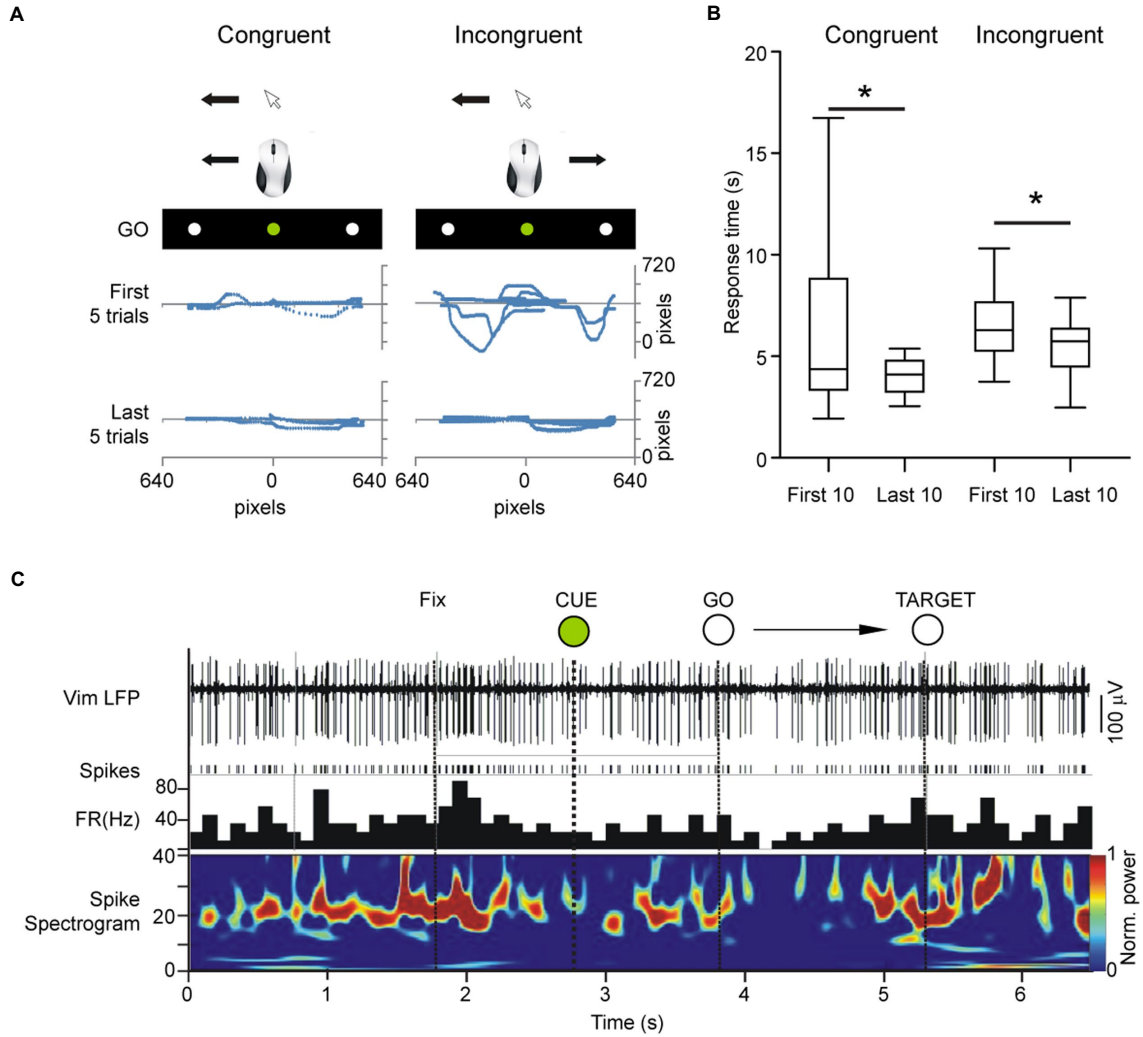
Center out task was shown to patients on an LCD monitor mounted over the patient in the operating room in a supine position and adjusted for full visibility. Due to restrictions imposed by the stereotactic frame, we opted to only investigate horizontally cued movements. The patient’s head was fixed toward the visual stimuli but moved the mouse on a flat surface without visual guidance of the hand/mouse movements. **(A)** Cursor traces of center-out movements in the first 5 and last 5 trials in the congruent and incongruent task, respectively. **(B)** Response times in incongruent versus congruent tracking tasks. Total time of response from center to periphery improved in both congruent and incongruent orientations. **(C)** Example local field potential of the Vim during the center-out task showing rhythmic multi-unit activity in the beta band and desynchronization of rhythmic firing during the task.

Following the identification of the nuclei and the stabilization of recordings, the patient was asked to use a computer mouse to perform a series of center-out hand movements that were guided by visual instructions presented on a computer monitor mounted above the surgical bed. This center-out task (Paradigm Experiments, Perception Research Systems Inc., United States) involved moving a mouse cursor on the computer screen from a central starting point to equidistant targets to the left or right of the central stimulus (baseline condition – congruent orientation) (Figure 1E). The patients moved the cursor to a central fixation point whereupon a preparatory “CUE” stimulus (white circle, called CUE hereafter) lasting 1 s was presented. The end of the 1 s CUE period was followed by an imperative “go” command (green circle, called GO hereafter). The central GO stimulus was followed by a peripheral target stimulus (white circle in the periphery, called TARGET hereafter), displayed in random order to the left or right of the center. Prior to the start of the experiments, the patients were shown a sample trial performed by the experimenter and were verbally informed of the instructional meaning of each stimulus: withhold movement during the 1 s interval between the CUE and the GO stimuli and move the cursor onto the peripheral TARGET after the TARGET appears. A total of 20 trials were conducted, one trial consisting of one center-out movement (CUE, GO, TARGET). The start time of the CUE stimulus and the arrival of the mouse cursor to the TARGET point was recorded concomitantly with spike and LFP recordings (Figure 2A).

To assess unfamiliar visuomotor coordination, the display was then horizontally inverted by digital manipulation so that leftward movements produced rightward deflections of the cursor on the screen and vice versa (experimental condition- incongruent orientation). The patients performed the same task as in the previous step, consisting of 20 trials, randomized for left/right peripheral targets. The incongruent condition represented an unfamiliar visuomotor relationship, comprised of normal motor output (i.e., rightward mouse movement) and unexpected visual feedback (i.e., leftward cursor movement). Mouse traces and center-out response times were recorded in order to assess reaction time and target accuracy (Figure 2A). Patients were not given practice trials in either congruent or incongruent orientations in order to preserve their unfamiliarity with the hand-eye coordination demands of the task.

### 2.4. Analysis

**Figure 3:**
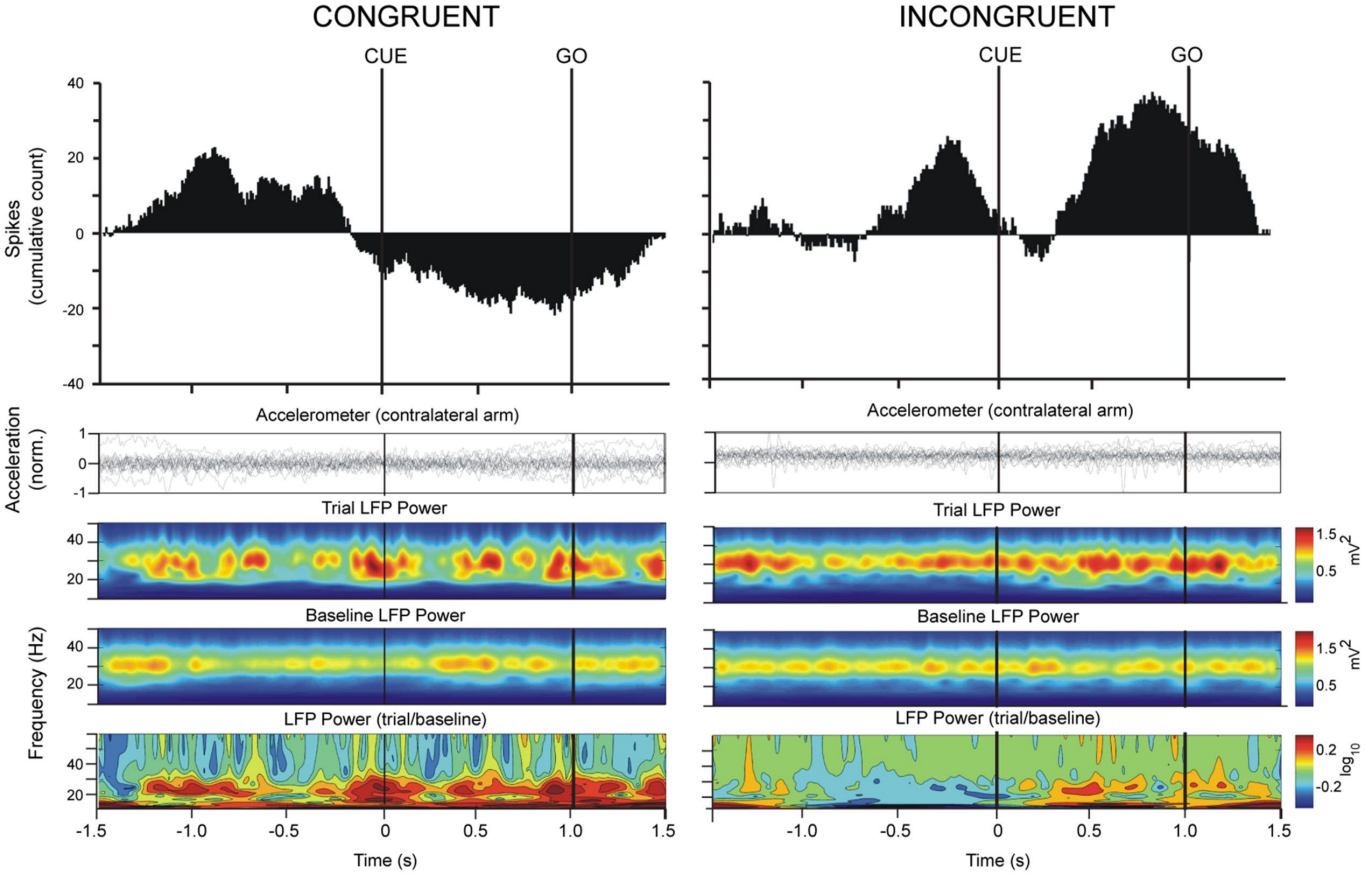
Simultaneously recorded spike and LFP data from 20 trials in an ET patient were averaged with respect to the CUE and GO triggers in congruent and incongruent paradigms. The top trace shows the cumulative sum of spiking activity of the Vim neuron and accelerometer traces are shown second from the top. The “trial LFP power” spectrogram shows average LFP power (filtered 13–30 Hz) from 20 trials referenced to the CUE trigger. The “baseline LFP power” plot was obtained by assigning 20 random triggers in the recording file and averaging the data over this trigger. The “LFP power (trial/baseline)” plot is the ratio of movement-related frequency-power changes to the baseline frequency power. Note that the power scale of the ratio is logarithmic such that a 0.1 increment reflects an increase by 25% of movement-related beta power in relation to baseline. During the performance of the center-out task in the congruent orientation, the increase of beta power during the preparatory phase occurred concomitantly with a decrease in neuronal firing of the Vim cell.

**Figure 4:**
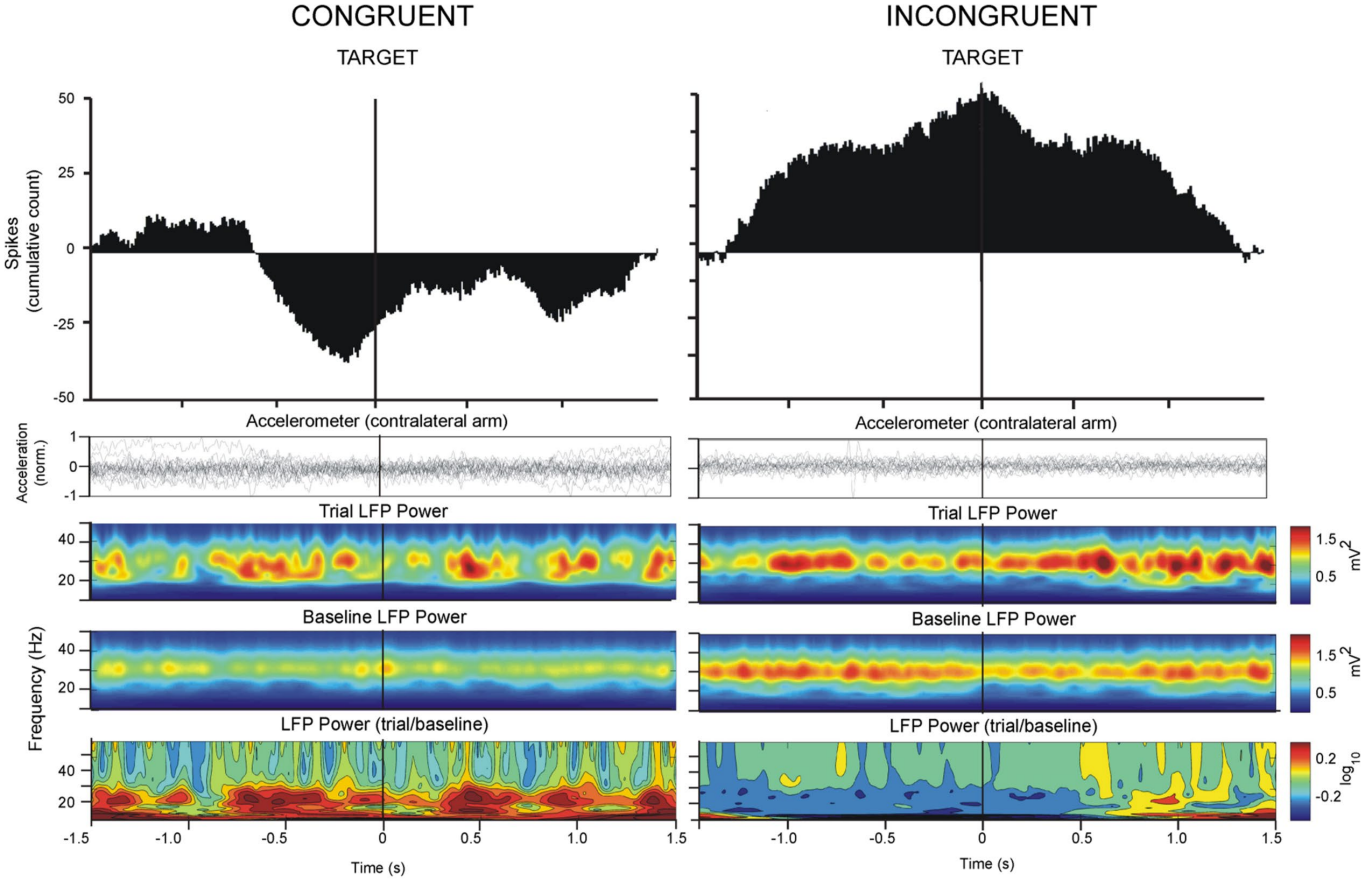
Analysis of LFP oscillations and spiking activity as in Figure 3, referenced to the TARGET trigger in congruent and incongruent paradigms. Vim neuronal firing decreased prior to TARGET approach in the congruent orientation. In the incongruent orientation, Vim firing increased on approach to TARGET concomitantly with a decrease in beta power.

The analysis of the data was centered around two epochs of the task: (1) the preparatory phase prior to the onset of movement (CUE → GO) and (2) the approach to the target (GO → TARGET) (Figure 2C). For epoch 1, recording segments starting 1.5 s prior to the onset of movement (GO) and ending 1.5 s after the onset of movement (GO) were selected for analysis. For epoch 2, recording segments starting 1.5 s prior to and ending 1.5 s after the arrival of the cursor to the peripheral target (TARGET) were selected (Figures 3, 4). The CUE-GO epochs were always 1 s in duration and were therefore not normalized. All data was referenced to CUE epoch at time 0. For TARGET, data was referenced to TARGET ±1.5 s. We did not consider the response time between GO-TARGET which was variable trial-to-trial and between patients. Single-unit activity was detected using template-matching tools in Spike2 (Cambridge Electronic Design, United Kingdom) and LFP data from both microelectrodes were bandpass filtered (IIR digital filter, 13–30 Hz). Briefly, fast signals (1–3 ms) were first detected as putative spikes according to a manually determined threshold that was adjusted based on signal-to-noise ratios in the recording. Next, template matching was carried out automatically according to “template widths” estimated by the Spike2 algorithm. Spike times were used to calculate the cumulative sum of the firing activity to outline trends in the firing rate during the performance of the task. Negative values indicate decreasing spiking activity and positive values indicate increasing spiking activity. The average cumulative spike count in the epoch of interest was used to compare spiking trends in the congruent versus incongruent tasks. LFP data were imported into MATLAB (version 6.5, The MathWorks, Natick, MA) for spectral analysis and peristimulus (GO or TARGET) time-frequency plots were obtained using in-house, custom scripts. Spectral power values from 13 to 30 Hz were imported into Excel 2007 (Microsoft Inc., Redmond, WA) and collapsed into a single average index (mean power over 13–30 Hz) in order to evaluate the mean change in beta power during the task.

### 2.5. Statistical analysis

Data were imported into Minitab (Minitab Inc., State College, PE) for statistical analysis. Continuous variables were compared using the *t*-test and ANOVA: (1) mean power values in epoch 1 (CUE → GO) during the congruent orientation on of the center-out task were compared to the same in the incongruent orientation and (2) mean power values in epoch 2 (GO → TARGET) during the congruent orientation were compared to that in the incongruent orientation. Similar comparisons were conducted for firing rates during the task.

## 3. Results

In both ET and PD groups, total times for performing the movement from CUE to TARGET were longer in the incongruent orientation in comparison to response times in the congruent orientation (*t*-test, *p* < 0.05). Response times were significantly lower in the last 10 trials in comparison to the first 10 trials in both the congruent and the incongruent condition (ANOVA, *p* < 0.05, Figure 2B).

### 3.1. Vim single-unit activity increased during incongruent center-out movements in ET

**Figure 5:**
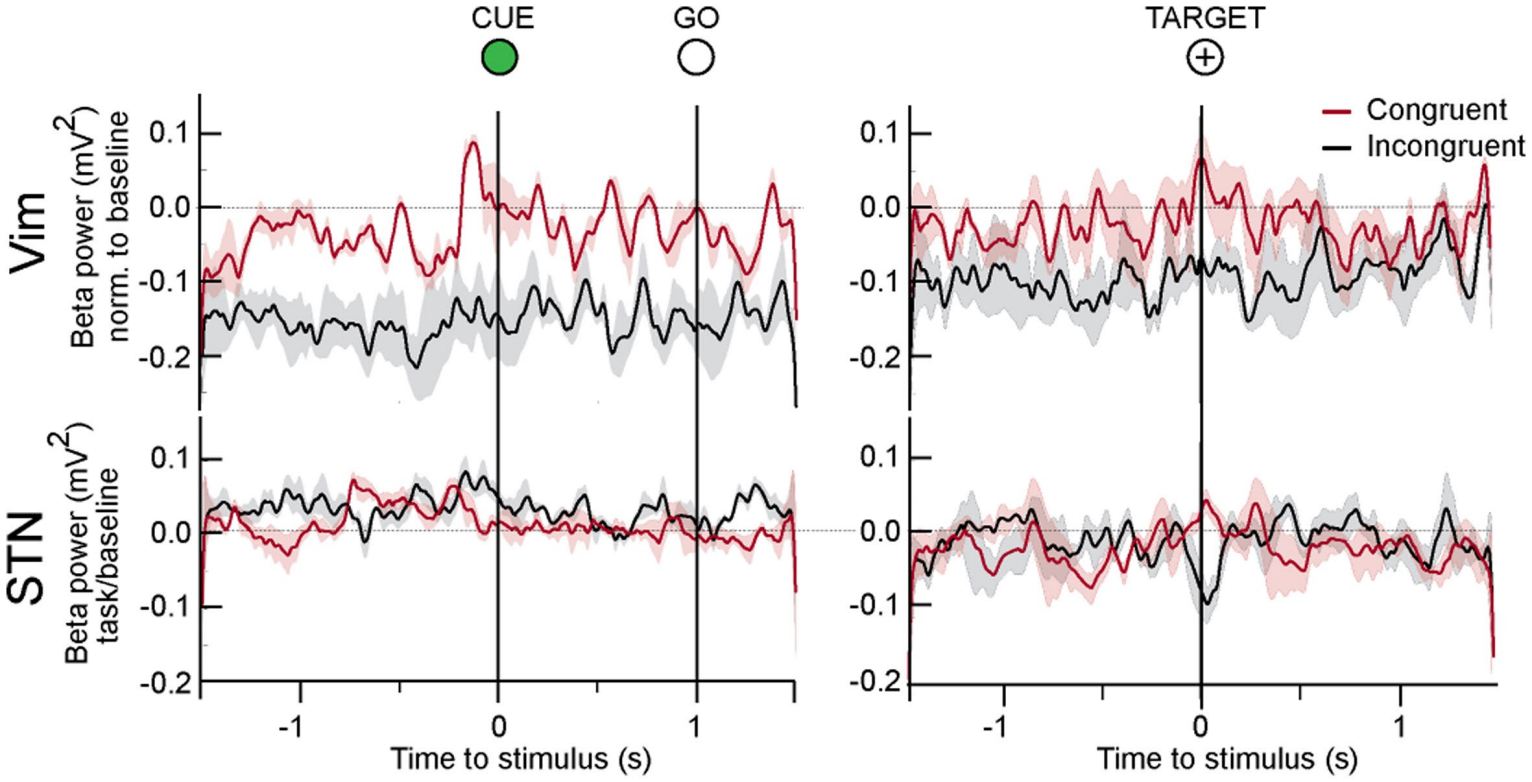
LFP responses to center out task in all patients. In the Vim of ET patients, beta activity was significantly lower during the whole duration of the incongruent center out task. In the movement preparation phase between CUE and GO, beta power increased in both tasks, although it was significantly lower in the incongruent condition. On approach to target, beta power was high in the congruent but lower in the incongruent orientation.

A total of 81 Vim single-units, recorded from 10 ET patients were analyzed (Figures 1D, F, G). The analysis of spike times from the Vim of ET patients showed that Vim single-unit activity increased during the incongruent center-out movements in comparison to the congruent orientation (epochs: 1. CUE ±1.5 s incongruent vs. congruent, *t*-test, *p* > 0.05; 2. TARGET ±1.5 s, incongruent vs. congruent, *t*-test, *p* > 0.05, Figures 3–5). Single-unit activity in the congruent orientation decreased during the preparatory phase (Figure 3A, CUE→GO versus 1 s before CUE, *p* < 0.05). In contrast, there was a significant increase in single-unit activity in the preparatory phase (CUE→GO) in comparison to the 1 s epoch before CUE (*t*-test, *p* < 0.05) in the incongruent orientation (Figure 3B). In the incongruent orientation, there was a significant buildup of activity leading up to the TARGET in comparison to the congruent orientation where spike activity decreased on approach to TARGET (Figure 4, TARGET ±250 ms, incongruent versus congruent, *t*-test, *p* < 0.05).

### 3.2. Vim beta power during incongruent center-out was lower than congruent orientation in ET

Spectral power analysis of the LFP from ET patients showed that Vim beta power was lower when patients performed the incongruent center-out task in comparison to the congruent orientation (Figure 5, epochs: CUE ±1.5 s and TARGET ±1.5 s, incongruent vs. congruent, *t*-test, *p* > 0.05) During both the congruent and incongruent center-out tasks, beta power increased around the approach to TARGET, compared to the period that preceded it (Figure 5, TARGET ±250 ms versus −750 to −250 ms prior to TARGET, *t*-test, *p* < 0.05).

### 3.3. STN beta power is unchanged during incongruent center-out movements in PD

We detected no significant difference in STN beta power in the CUE-GO epoch between the congruent and incongruent orientations (Figure 5). A transient decrease in beta power was detected around the TARGET in the incongruent paradigm that was significantly different from the congruent condition (ANOVA, *p* < 0.01).

## 4. Discussion

In this study, we found that Vim beta oscillations of ET patients are suppressed during the performance of an unfamiliar task that required attentive visuomotor coordination, as tested by the incongruent center-out task. In the preparatory phase prior to the initiation of the movement in the incongruent center-out task, beta power in the Vim increased in concert with a small increase in spiking activity. On the approach to target in the incongruent center-out movement, Vim beta oscillations were significantly decreased in concert with a large buildup of spiking activity. In contrast, the power of STN beta oscillations was not significantly changed between the incongruent and congruent orientations of the center-out task. This suggests that the beta activity in motor thalamus is not related to beta from the basal ganglia but rather cerebellar beta oscillations.

The decreased beta power during the unfamiliar, visuomotor mismatch task suggests a relationship between thalamic beta oscillations and the adaptive processing of visuomotor information. In the cortex, the magnitude of beta desynchronization depends on the subject’s familiarity with the motor task being performed, suggesting that beta desynchronization may reflect the level of expertise in learned movements (Kilavik et al., 2013). In the early stages of learning, when a subject is unfamiliar with the sensorimotor dynamics of the task, large beta desynchronization is correlated with superior performance (Pollok et al., 2014). The beta rhythm is thought to facilitate such motor learning by providing a temporal link between sensory and motor centers (Bibbig et al., 2002), allowing the brain to evaluate the sensory consequences of a movement in real time. Thus, a familiar task such as the manipulation of a computer mouse may produce only mild beta desynchronization considering that the mouse cursor (visual feedback) responds predictably to the movement of the mouse itself (motor command). However, unexpected sensory feedback imposed by the mismatched visuomotor relationship desynchronizes beta, likely as a mechanism for adaptive visuomotor integration. The post-movement beta rebound has also been suggested to reinforce existing motor states and steady motor output (Engel and Fries, 2010; Tan et al., 2016) and appears to be involved in the processing of movement-related sensory afference (Alegre et al., 2008) and in errors related to the completed movement (Tan et al., 2014).

### 4.1. Vim beta oscillations and Vim firing rates

The suppression of Vim beta power during the incongruent center-out task and the concomitant increase in spiking suggests a relationship between beta amplitude and firing rate. Previous work has demonstrated that the cerebellum is activated during unfamiliar hand-eye relationships such as that imposed by prism goggles (Weiner et al., 1983) or perturbation by force fields (Chen et al., 2006). This increased firing may be related to error signaling neurons that fire vigorously when there is a discrepancy between the desired target and the actual hand or eye position and are silent once the target is attained [for review see (Optican and Robinson, 1980; Optican and Pretegiani, 2017)]. In the Vim, Chen et al. (2006) showed that neurons change the timing of their firing when the subject is engaged in visual rotation task whereby the target at the end of the movement is shifted. The modulation of beta power with respect to this shift may contribute to the timing and predictive aspect of this movement and secondly, low beta power may facilitate throughput from the cerebellum. During the familiar center-out movement, beta power remained high in the Vim while spiking activity was low, with beta possibly acting as a suppressive signal to cerebellar input. Crandall et al. (2015) have shown that cortically-driven oscillations in the thalamus suppress thalamic excitability to peripheral input and may thus act as a “dynamic switch” that controls the transmission of information to the cortex. Similarly, during sleep, when rhythmic spindle activity predominates in the thalamus, peripheral throughput to the cortex is low in fidelity (McCormick and Feeser, 1990). In this regard, beta oscillations in the thalamus may serve a similar gating function by suppressing behaviorally irrelevant information while maintaining the existing representation of the motor program within the thalamocortical circuit. Their attenuation during novel and unexpected states in the periphery may promote the transmission of new information to the cortex.

The relationship between beta amplitude and firing rate is still controversial. Either beta ERD promotes Vim firing during cerebellar activation or beta ERD is a consequence of individual units coming out of the population firing at a synchronous lower rate. Some authors have provided evidence for an inverse or a direct 1–1 relationship or both, and no relationship at all (see Confais et al., 2020). The variable results may reflect the fact these are different signals. The LFP is a summation of extracellular currents at a given point and synaptic currents are a major (but not exclusive) component and so reflect input to a nucleus, whereas spiking is the output of the nucleus (Buzsaki and Wang, 2012). In an analysis of cell-LFP beta coherence in pairs of human STN neurons, only 35% had a significant coherent relation to the beta LFP (Alavi et al., 2013) but similar studies have not been done on cerebellar thalamic beta. It is possible that different nuclei in the basal ganglia thalamocortical network have different cell firing to beta-LFP relationships.

The observation that beta power was not different during the incongruent and congruent orientation in PD patients may reflect the oscillatory pathology of PD patients (Levy et al., 2002; Pogosyan et al., 2010). As noted, increased beta synchrony is associated with parkinsonism and as such, the ability to suppress beta may be affected in this group of patients.

There are several limitations to the study. Comparison of beta in STN and Vim may be confounded by the pathology in both groups of patients, and the recordings are invasive, so it is not possible to compare with controls. The task was difficult for patients to conduct, especially in the 12 h off medication condition of the PD group so the single unit population was limited, whereas the beta could be measured in all patients. Further studies would be required to obtain more single unit data in a larger patient cohort.

## Data availability statement

The raw data supporting the conclusions of this article will be made available by the authors, without undue reservation.

## Ethics statement

The studies involving human participants were reviewed and approved by Research Ethics Board of University Health Network. The patients/participants provided their written informed consent to participate in this study.

## Author contributions

DB and WH: study design and execution. SK, MH, AML, and ALR: surgical procedure and patient care. DB: preparation of first draft. WH and DB: revisions. SK, MH, ALR, and AML: final approval of manuscript. All authors contributed to the article and approved the submitted version.

## Funding

This work was supported by DMRF USA.

## Conflict of interest

The authors declare that the research was conducted in the absence of any commercial or financial relationships that could be construed as a potential conflict of interest.

## Publisher’s note

All claims expressed in this article are solely those of the authors and do not necessarily represent those of their affiliated organizations, or those of the publisher, the editors and the reviewers. Any product that may be evaluated in this article, or claim that may be made by its manufacturer, is not guaranteed or endorsed by the publisher.
